# The moral injury of zeeple‐zoople

**DOI:** 10.1111/acem.70025

**Published:** 2025-03-19

**Authors:** Nike Izmaylov, Michelle Izmaylov

**Affiliations:** ^1^ Vanderbilt University Medical Center Nashville Tennessee USA

I walk into an auditorium for grand rounds and find evidence of zeeple‐zoople everywhere: its taste from an attending's stale bagel as she hurriedly rounds on an overcapped patient census, its smell in the splashed backflow of a hasty diagnostic paracentesis on a nurse practitioner's scrubs while he glances through signout for an admission bolus, its sound behind a resident's gritted teeth as she places orders on her phone instead of having a few minutes to pay attention to an educational event.

The speaker, a physician associated with an Ivy League medical school, arrives for the presentation. The attending, nurse practitioner, and resident look up.

This speaker claims to have the solution for burnout in medicine.

The speaker expresses concern about national burnout statistics. The pain of hearing those statistics presses a knife between the attending's ribs, a cutting pain. Her friends from her medical school and residency program comprise some of those statistics.

The presenter discusses the framing of “moral injury”: when a person feels *systematically* unable to do what they perceive as morally right, which distresses the person in a manner more than *individual* burnout. The attending sees the resident set aside her phone, now paying attention to each word spoken, these words that seem to address the hurt that many clinicians feel each day at work.

But this turns to a discussion of what clinicians perceive to be moral injury—and what language clinicians *should* use to describe their situation.

The attending listens, though she is not certain how this detour is relevant to solving the weight of work on her shoulders. She sees the nurse practitioner frown, too.

The presenter provides a specific example: an emergency department (ED) attending was working alone one night during a busy shift. A patient with mental illness and documented disruptive behavior became combative with staff already overwhelmed with their workload. The physician recognized that the ED lacked staffing capabilities to provide care for all these patients. He assessed the combative woman, determined she wasn't suicidal, and wheeled her out of the ED—but was not able to provide all the care he thought the patient needed. The physician described this as a moral injury resulting from systemic factors such as the ED's insufficient staffing despite each staff member working intensely.

The presenter argues: this is *not* a moral injury.

The attending listens. She feels the sharpness of the blade pressed against her ribs.

Instead, the presenter argues, the ED physician's perception that the system failed him—that he suffered a moral injury—is using the system to evade his own failures. The attending hears what is implied: the presenter thinks the ED physician was making excuses instead of just working harder to accomplish everything demanded of him.

Additional examples are presented: the medical student expected to stay late to obtain medical records, the resident told to assess a new patient at the end of a shift. The presenter argues these are not a moral injury—these are just the responsibility of the individual who signed up to be a clinician, regardless of how feasible the work is in the circumstances.

When these incidents pile up to make clinicians feel burned out, the presenter argues this does not equal depression. The nurse might have to miss her friend's wedding, and the social worker might not see his daughter for most of the week. But this *should* not result in depression.

“Depression” is a serious matter, worthy of outside help; “burnout” is merely an expected manifestation of a clinician's work. The solution to “depression” might require therapy or medications. The solution to “burnout,” according to the speaker, is to change our perspectives on medicine, to honor and worship the privilege of our employment.

Those who mistakenly refer to their “burnout” as “depression,” the presenter continues, are leveraging a “*real*” problem to justify their work dissatisfaction in a culture that devalues the “profession.”

The speaker presents this solution: we must confront the problems with individuals who shrug rather than do the necessary work. The presenter argues that if physicians viewed medicine as a calling and a privilege, then they would be willing to perform the individual work themselves and assist patients more. The physician assistant, resident, and ED attending should have known what they were getting into with a career in medicine.

Essentially, the solution to burnout is to convince clinicians to work harder instead of blaming the system for their own failures.

When the attending leaves the auditorium after the presentation, she stands for a while in the hallway. The roses she thought she would find in this grand rounds lecture have all turned to the threat of thorns, the sharpness of the argument she heard felt like a knife through her body.

Is this the status of the medical system? Are clinicians quitting because they are just shifting blame to a system? Are ED physicians just not willing to step up to the calling of medicine, when they are on the front lines dealing with patients that many hospital doctors never would? What about the internists who are quitting as they are given increasingly larger patient censuses and administrative burdens such as documentation demands or the nurses who are resigning as increasing ratios of patients‐to‐nurses leaves them hurrying away from a worried patient to provide medications to another patient? How can intensifying individual effort solve challenges such as multiple simultaneous patient decompensations needing the assessment of the single clinician staffing a service?

Is the solution to all of this found in working harder and examining the language used to assign blame?

The language we use certainly matters. Studies have found that patients with substance use disorders have improved outcomes when language removes the blame from their shoulders. There has been no convincing evidence that telling patients they are responsible for their own addictions improves their ability to recover. Nor have I found convincing evidence that telling overwhelmed clinicians that working even harder will fix their mental burdens, whether or not they have been “diagnosed” with depression. Then why is this the solution we present for clinicians already finding it difficult to get through each day in medicine?

The attending goes back to assessing her patients. She does the work necessary, stays late, skips plans with her friends. This is what is expected of her. Previously in her career, the attending would have protested the content of the grand rounds presentation to her leadership. But she has learned not to protest—despite the pain of the blade in her side—because most complaints are dismissed without altering working conditions or transformed into "an opportunity for individual feedback," the blame turned back on her and *her* inability to complete documentation for ever‐increasing patient censuses and *her* inability to complete increasing amounts of consults and *her* inability to complete documentation queries that do not impact her patients' care, continuous reminders of *you signed up for “this,”* when “*this*” is—

—all of a clinician's efforts for patient care are considered not as significant as her documentation, when “*this*” is—

—more patients to assess in an hour than she can possibly see in the whole day, when “*this*” is—

—a clinician standing by a busy street and debating whether stepping off the curb would be preferable to stepping into another shift.

And yet “*this*” is not improving at a rate most clinicians find acceptable, “*this*” seems to continue to get worse, and all of “*this*” pushes so many clinicians into leaving medicine by any means necessary.

The attending attempts to be more efficient, to be more productive, to increase how much work she does. When she continues to not meet expectations despite her individual effort, the attending considers turning the pain of that blade tangible.

For all the clinicians whose suffering is described with statistics about burnout and suicide, I protest the argument of that grand rounds lecture.

The semantics of whether it is “moral injury” or “laziness,” “depression” or “burnout,” that pushes clinicians to quit and commit suicide must not be prioritized ahead of concrete systemic improvements. The speaker argues that individuals must step up despite the failures of the system. But this is not sustainable.

When systemic changes can decrease the number of people quitting or killing themselves, then they are worth pursuing, no matter where the problems originate.

From my perspective, it does not matter whether you call the problems in medicine “burnout,” “depression,” or “zeeple‐zoople.” It matters that “zeeple‐zoople” is causing us to quit and to kill ourselves. We value productivity in medicine; then, let us turn the energy expended to assign blame into productive energy to find solutions. Let us redirect those efforts to divide us into efforts to unite us, a collective effort to improve the system for ourselves, our colleagues, and our patients, all of us stepping up to contribute solutions to clinician burnout and suicide in medicine.

## CONFLICT OF INTEREST STATEMENT

The authors declare no conflicts of interest.

## Data Availability

Data sharing not applicable to this article as no datasets were generated or analysed during the current study.

